# Disentangling a complex nationwide *Salmonella* Dublin outbreak associated with raw-milk cheese consumption, France, 2015 to 2016

**DOI:** 10.2807/1560-7917.ES.2019.24.3.1700703

**Published:** 2019-01-17

**Authors:** Aymeric Ung, Amrish Y. Baidjoe, Dieter Van Cauteren, Nizar Fawal, Laetitia Fabre, Caroline Guerrisi, Kostas Danis, Anne Morand, Marie-Pierre Donguy, Etienne Lucas, Louise Rossignol, Sophie Lefèvre, Marie-Léone Vignaud, Sabrina Cadel-Six, Renaud Lailler, Nathalie Jourdan-Da Silva, Simon Le Hello

**Affiliations:** 1 Santé publique France (SpFrance), the French national public health agency, Saint-Maurice, France; 2 European Programme for Intervention Epidemiology Training (EPIET), European Centre of Disease Prevention and Control (ECDC), Stockholm, Sweden; 3 These authors contributed equally to this article and share first authorship; 4 European Programme for Public Health Microbiology Training (EUPHEM), European Centre of Disease Prevention and Control (ECDC), Stockholm, Sweden; 5 Institut Pasteur, Enteric Bacterial Pathogens Unit, National Reference Center (NRC) for *E. coli*, *Shigella* and *Salmonella*, Paris, France; 6 Sorbonne Université, UPMC, INSERM, Institut Pierre Louis d’épidémiologie et de Santé Publique, IPLESP, Paris, France; 7 French Directorate General for Food (DGAL), Ministry of Agriculture and Food, Paris, France; 8 Université Paris-Est, French Agency for Food, Environmental and Occupational Health and Safety (ANSES), Laboratory for Food Safety, Maisons-Alfort, France; 9 These authors contributed equally to this article and share last authorship

**Keywords:** *Salmonella* Dublin, outbreaks, raw-milk, cheese, France, WGS

## Abstract

On 18 January 2016, the French National Reference Centre for *Salmonella* reported to Santé publique France an excess of *Salmonella enterica* serotype Dublin (*S.* Dublin) infections. We investigated to identify the source of infection and implement control measures. Whole genome sequencing (WGS) and multilocus variable-number tandem repeat analysis (MLVA) were performed to identify microbiological clusters and links among cases, animal and food sources. Clusters were defined as isolates with less than 15 single nucleotide polymorphisms determined by WGS and/or with identical MLVA pattern. We compared different clusters of cases with other cases (case–case study) and controls recruited from a web-based cohort (case–control study) in terms of food consumption. We interviewed 63/83 (76%) cases; 2,914 controls completed a questionnaire. Both studies’ findings indicated that successive *S.* Dublin outbreaks from different sources had occurred between November 2015 and March 2016. In the case–control study, cases of distinct WGS clusters were more likely to have consumed Morbier (adjusted odds ratio (aOR): 14; 95% confidence interval (CI): 4.8–42) or Vacherin Mont d’Or (aOR: 27; 95% CI: 6.8–105), two bovine raw-milk cheeses. Based on these results, the Ministry of Agriculture launched a reinforced control plan for processing plants of raw-milk cheeses in the production region, to prevent future outbreaks.

## Background

Nontyphoidal *Salmonella* is a main cause of bacterial food-borne infection in Europe [1,2]. The majority of human infections is caused by a limited number of *Salmonella* serotypes among the 2,600 described to date [3,4]. *Salmonella enterica* serotype Dublin (*S*. Dublin) is particularly invasive in humans and more often leads to severe disease and higher mortality rates compared with other serotypes [4-7]. *S*. Dublin is host-adapted to bovines and is frequently isolated from cattle, with raw milk or raw-milk cheeses as a typical vehicle for food-borne outbreaks [8,9]. In 2012, a major *S*. Dublin outbreak occurred in France, with 103 cases linked to Saint-Nectaire (bovine raw-milk cheese) consumption [10,11]. In 2015, 34 *S*. Dublin cases were reported linked to the consumption of Reblochon (bovine raw-milk cheese) (data not shown; Santé publique France).

In France, the National Reference Center for *Salmonella* (NRC) and the French Agency for Food, Environmental and Occupational Health and Safety (ANSES) routinely collect and serotype human and non-human *Salmonella* isolates, respectively [12-14], using the Kauffmann–White–Le Minor scheme [3]. The *S.* Dublin isolates collected are frequently susceptible to all antibiotics and show an indistinguishable pulsed-field gel electrophoresis (PFGE) pattern. To better distinguish *S*. Dublin isolates, multilocus variable-number tandem repeat analysis (MLVA) has recently been used for surveillance and outbreak investigations [11,15]. Moreover, whole genome sequencing (WGS) of *Salmonella* has been shown to discriminate between closely related isolates of *S*. Dublin [16,17].

### Outbreak detection

On 18 January 2016, the French NRC reported to Santé publique France (SpFrance, the French national public health agency) an excess of *S*. Dublin infections across the country, with 37 *S*. Dublin isolates identified between mid-November 2015 and mid-January 2016, compared with 10 *S*. Dublin isolates during the same period in the two previous years. An outbreak investigation team with experts from SpFrance, NRC, ANSES and the French Directorate General for Food (DGAL) launched extensive epidemiological, microbiological and food investigations to confirm the outbreak, identify the vehicle of transmission and propose appropriate control measures.

## Methods

We carried out both epidemiological and microbiological investigations on subsets of *S*. Dublin cases and isolates, respectively.

### Microbiological investigations

#### *Salmonella* Dublin isolates

**Figure 1:**
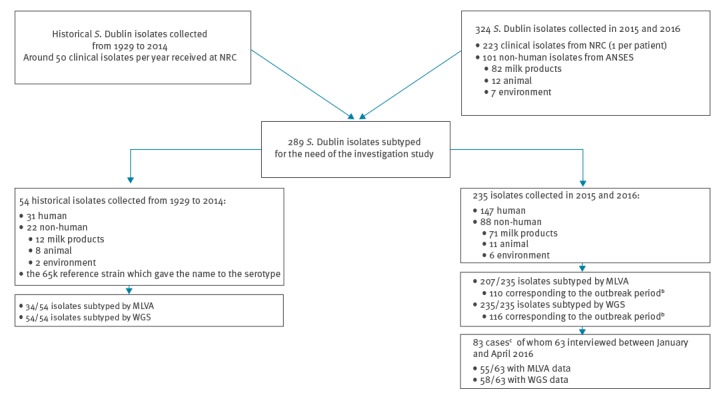
Flowchart of human and non-human *Salmonella* Dublin isolates^a^ subtyped and included in the study, France, 2015–2016 (n = 289)

ANSES: French Agency for Food, Environmental and Occupational Health and Safety; MLVA: multilocus variable-number tandem repeat analysis; NRC: national reference center; WGS: whole genome sequencing.

^a^ While the study was conducted between 2015 and 2016, the subtyped isolates also included historical strains isolated between 1929 and 2014.

^b^ The outbreak period is from 17 November 2015 to 11 March 2016.

^c^ Case definition: residents in mainland France with a *S*. Dublin infection reported to the NRC between 17 November 2015 and 11 March 2016.

During the years 2015 and 2016, a total of 324 *S.* Dublin isolates were collected, 223 from clinical NRC isolates (108 in 2015 and 115 in 2016) and 101 from non-human ANSES isolates (62 and 39, in each year respectively). Of those, a total of 235, including 147 (83 in 2015 and 64 in 2016) clinical and 88 (62 and 26, respectively) non-human isolates, were extensively studied by WGS and/or MLVA. We also analysed 54 ‘historical’ subtyped *S.* Dublin isolates collected between 1929 and 2014, 31 from humans and 22 from non-human specimens, as well as the human isolate which gave the name to the serotype in 1929 (number 65k) [18]. In total, we included 289 isolates in this study (Figure 1).

#### Whole genome sequencing

The NRC used WGS to subtype all the 289 isolates of which 116 corresponded to the outbreak period from November 2015 to March 2016. High-throughput genome sequencing was carried out at the ‘Plateforme de microbiologie mutualisée’ (P2M) of the Pasteur International Bioresources network (Institut Pasteur, Paris, France). After extraction with the MagNA Pure 96 System (Roche, Basel, Switzerland), DNA was further processed for sequencing with Illumina systems (libraries using the Nextera XT DNA Library Prep kit and the sequencing with the NextSeq 500 system) generating 100 to 146 bp paired-end reads. Reads were trimmed and assembled as previously described [19]. Genomic data as multilocus sequence typing (MLST) type and resistance genes were detected from assembled sequences using web-tools (http://www.genomicepidemiology.org/). For each isolate, the paired-end reads were aligned against the *S.* Dublin str. 3246 reference genome (GenBank accession number: CM001151.1) using Bowtie2 with default parameters [20]. A core-genome multi-alignment of assembled genomes was also done using Harvest v1.0.1 f ParSNP function [21]. For each approach, the resulting single nucleotide polymorphisms (SNPs) were concatenated to generate a filtered multiple alignment that was used as input for the construction of a phylogenetic tree using Molecular Evolutionary Genetics Analysis (MEGA)6 [22] with a maximum-likelihood (ML) approach. The final trees were visualised in the interactive Tree Of Life [23]. All reads generated in this study have been deposited in project PRJEB28817.

#### Multilocus variable-number tandem repeat analysis

ANSES used MLVA as described elsewhere [11], to analyse 241 isolates (including 148 human and 93 non-human), of which 110 corresponded to the outbreak period. The measured lengths for each fragment were obtained using an ABI3500 capillary electrophoresis system (Applied Biosystems, France). Data were imported into GeneMapper software (Applied Biosystems, France) where each fragment was identified according to colour and size. A normalisation of the results was done with the free access MLVA_Normalizer software [24].

### Epidemiological investigation

#### Case definition

We defined cases as residents in mainland France with a *S.* Dublin infection reported to the NRC between 17 November 2015 and 11 March 2016, without travel history outside of France within 7 days prior symptom onset and without history of *S.* Dublin infection before 17 November 2015 (i.e. no *S*. Dublin strain ever isolated and received at the NRC before 17 November 2015).

#### Cluster definition

We defined a cluster as isolate sequences with < 15 SNP divergence obtained by core-genome comparison and/or with identical MLVA pattern. Among these clusters, we also defined subclusters as isolate sequences having < 5 intra SNPs.

### Study design

We compared cases belonging to a specific cluster/subcluster with other cases belonging to all other clusters/subclusters in a case–case study and with controls in a case–control study. For the case–control study, controls were recruited from a cohort of individuals with children and adults registered on GrippeNet.fr (https://www.grippenet.fr), an online population-based surveillance system for influenza-like illness [25,26]. During winter 2015/16, 6,515 participants reported online the presence or absence of basic symptoms on a weekly basis using a list of 19 predefined symptoms commonly or rarely related to influenza. We excluded controls reporting travel abroad during the *Salmonella* outbreak period and those with digestive symptoms.

### Data collection

Epidemiologists from the regional offices of SpFrance interviewed the cases by telephone using a trawling questionnaire on clinical symptoms, medical history, detailed food consumption, contact with other persons experiencing diarrhoea, travel history and contact with animals. Loyalty card information was collected to trace-back supermarket purchases.

On week 8 (22–28 February), 2016, controls from the GrippeNet.fr cohort received a web link to complete an online questionnaire about health, travel history and food consumption during 11–17 January 2016 (a 7-day ordinary non-festive period).

### Statistical analyses

We calculated proportions, using the number of non-missing values as denominators. For the case–case study, we calculated crude odds ratios (OR). For the case–control study, we calculated adjusted odds ratios (aOR) for age and sex using multivariable logistic regression. The initial regression models included age, sex and food items consumed by at least 50% of the cases. We performed this analysis for WGS clusters/subclusters and MLVA clusters with at least 10 cases. We used STATA version 12.0 (Stata Corporation, Texas, United States) for this analysis.

### Ethical considerations

The study was approved by the French Commission for Data Protection (Commission Nationale de l’Informatique et des Libertés). Interviewees or next of kin provided verbal consent. Only anonymised data were analysed and used for the purpose of the study.

### Food production chain and animal trace-back investigations

DGAL conducted a trace-back investigation on potential contaminated products identified by the epidemiological investigations. Points of purchase, like supermarkets or cheese retailers, were reported by cases. Where possible, customer loyalty card numbers were used to identify the exact point of purchase and product batch numbers and specific production facilities. The trace-back investigation was conducted for a retrospective period of up to a month before symptom onset of given cases, if date of onset was available, or if not, up to two months before the date of isolation of *S*. Dublin in the patient specimen. Suspected food products were tested for *Salmonella* if food samples were available.

## Results

### Description of cases

Between 17 November 2015 and 11 March 2016, 83 cases were identified. Median age was 70 years (range: 1–94), 44 (53%) were female and respondents originated from 12/13 regions in mainland France, with 19 (23%) cases coming from the Bourgogne-Franche-Comté region (Table 1). *S.* Dublin was isolated from blood (n = 39; 47%) but also from stool (25; 30%), urine (11; 13%), and other samples (8; 10%). Ten (12%) deaths were reported with no information available on the cause of death. Questionnaires were not completed for 20 cases (9 deaths, 7 unreachable, 4 refusals), leading to 63 (76%) cases included in further analyses.

**Table 1:** Characteristics of *Salmonella* Dublin outbreak cases, France, November 2015–March 2016 (n =  83, including 63 interviewed cases)

Characteristics of cases	Category	n	N^a^	%
**All cases (n = 83)**				
Sex	Female	44	83	53
Age	1–17	6	83	7
	18–44	10	83	12
	45–64	19	83	23
	65–84	31	83	37
	85–94	17	83	21
Region of residence	Auvergne-Rhône-Alpes	8	83	10
	Bourgogne-Franche-Comté	19	83	23
	Bretagne	6	83	7
	Grand-Est	6	83	7
	Haut-de-France	7	83	8
	Île-de-France	9	83	11
	Nouvelle-Aquitaine	9	83	11
	Pays-de-la-Loire	7	83	8
	Other^b^	12	83	15
Type of human sample	Blood	39	83	47
	Stool	25	83	30
	Urine	11	83	13
	Articular fluid	3	83	4
	Pus	2	83	2
	Other	3	83	4
Deceased	Yes^c^	10	83	12
**Interviewed cases (n = 63)**				
Comorbidities	Yes^d^	39	59	NA
Clinical symptoms^e^	Fever^f^	38	55	NA
	Nausea	15	45	NA
	Vomiting	19	51	NA
	Abdominal pain	27	47	NA
	Diarrhoea	30	56	NA
	Blood in faeces	5	38	NA
Hospitalisation	Yes	41	60	68

NA: Not applicable due to low figure in the denominator (< 60).

^a^ Number of cases for which the information was available.

^b^ Centre Val-de-Loire, Normandie, Occitanie and Provence-Alpes-Côte d’Azur.

^c^ Information provided by the National Reference Center for *Salmonella* (NRC), without confirmation that cause of death was attributable to *S*. Dublin infection.

^d^ Asthma, cancer, cardiac problems, diabetes, renal failure.

^e^ Cases could have more than one clinical symptom.

^f^ Fever was defined as body temperature > 38°C or perception of having fever.

Among these cases, 39 of 59 with available information reported having pre-existing chronic medical conditions (asthma, cancer, cardiac problems, diabetes, renal failure). Most frequently reported symptoms included fever (either reported as a measurement of body temperature > 38°C or reported as a perception; 38/55), abdominal pain (27/47) and diarrhoea (30/56). Among cases with data on hospitalisation, 68% (41/60) were hospitalised. 

**Figure 2:**
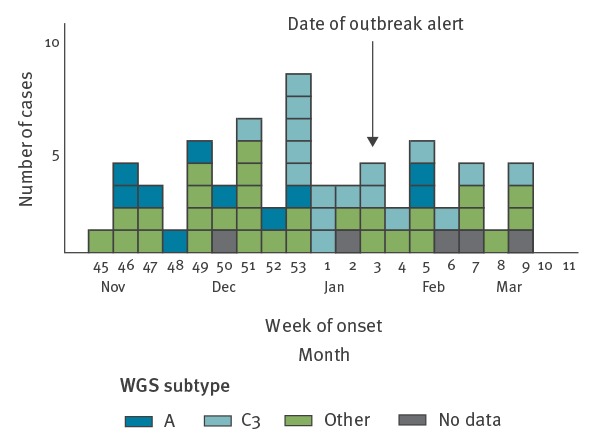
Number of cases by reported^a^ date of onset of symptoms^b^, *Salmonella* Dublin outbreak, France, November 2015–March 2016 (n = 83^b^)

The number of cases peaked during week 53 (28 December–3 January) (Figure 2).

WGS: whole genome sequencing.

^a^ 55 cases reported a precise day or week of onset and six cases reported a vague period of symptom onset time (within several weeks); we then recorded the last week of that period as the week of onset of symptoms.

^b^ For 22 cases, no date of onset was recorded; of these, we did not have a completed questionnaire for 20.

### Description of controls

Among the 6,200 GrippeNet.fr participants (i.e. controls) who could be reached, 2,914 (47%) completed the questionnaire. Of those, 2,690 (92%) did this within 2 days; 1,916 (66%) were female; median age was 56 years (range: 2–90). The whole process, i.e. from the beginning of the recruitment of the controls to the end of data collection, took 12 days.

There were significant differences between cases and controls in terms of proportion of female (53% in cases vs 66% in controls; p value = 0.01) and age (58% of cases vs 29% of controls were > 65 years-old; p value = 0.00).

### Microbiological findings

Among 241 MLVA performed for *S.* Dublin isolates, 49 different MLVA patterns were obtained and among these, seven gathered 10 or more isolates (Table 2).

**Table 2:** Number of subtyped human and non-human *Salmonella* Dublin isolates by MLVA patterns (rows) and whole genome sequencing clusters (columns) included in the study, France, 2015–2016^a^ (n  = 289)

MLVA	WGS							Total
	A	B	C3	C_other_	F	K	Other^b^	
**15–8-10–7-5–3**	0	0	0	1	19^c^	0	0	20
**17–8-10–7-5–4**	0	9^d^	0	6	0	0	0	15
**18–8-10–7-5–4**	0	14^e^	28	3	0	0	2	47
**19–8-10–7-5–3**	0	0	0	22^f^	0	0	0	22
**19–8-10–7-5–4**	1	4	1	18^g^	0	0	3	27
**20–8-10–7-5–3**	0	0	0	10	0	0	0	10
**20–8-10–7-5–4**	15	1	0	0	0	0	1	17
**Other^h^**	0	5	1	11	15	11	40	83
**Missing**	2	2	5	17	4	0	18	48
**Total**	18	35	35	88	38	11	64	289

MLVA: multilocus variable-number tandem repeat analysis; WGS: whole genome sequencing.

^a^ While the study was conducted between 2015 and 2016, the subtyped isolates included historical strains isolated between 1929 and 2014.

^b^ There were 23 other WGS subtypes.

^c^ Among these 19, 13 were associated with WGS subcluster F4.

^d^ All nine isolates with MLVA 17–8-10–7-5–4 pattern were associated with WGS subcluster B3.

^e^ Eight isolates with MLVA 18–8-10–7-5–4 pattern were associated with WGS subcluster B4.

^f^ Fourteen isolates with MLVA 19–8-10–7-5–3 pattern were associated with WGS subcluster C8.

^g^ Eight isolates with MLVA 19–8-10–7-5–4 pattern were associated with WGS subcluster C6.

^h^ There were 42 other MLVA patterns.

**Figure 3:**
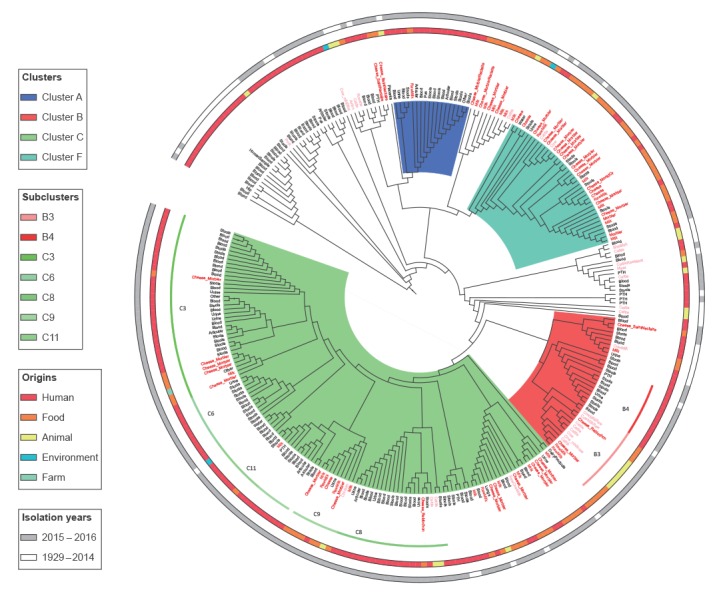
Phylogenetic analysis of all the subtyped human and non-human *Salmonella* Dublin isolates included in the study that were whole genome sequenced, France, 2015–2016^a^ (n = 289)

^a^ While the study was conducted between 2015 and 2016, the subtyped isolates included historical strains isolated between 1929 and 2014.

The WGS analysis of all the 289 *S*. Dublin isolates, including historical ones, indicated a unique MLST, ST10 that had previously been related to serotype Dublin in both the literature and public databases (https://enterobase.warwick.ac.uk/) [27]. As the mapping against the *S*. Dublin str. 3246 reference genome (GenBank accession number: CM001151.1) showed high divergence with 19,213 SNPs, the core-genome multi-alignment of assembled genomes using ParSNP function was preferred. A generated matrix file revealed that maximum divergence for all isolates sequenced during the outbreak including the reference strain was 791 SNPs suggesting a relative homogeneous population of *S*. Dublin isolates that were circulating in France. We identified 28 different clusters with < 15 SNPs, including five clusters with > 10 isolates (clusters A, B, C, F and K). Three of those, A, B and C, accounted for the majority of human (70%, 125/179) and non-human isolates (46%, 51/110) (Figure 3).

A total of 18 isolates (17 human and 1 non-human) with date of isolation between October 2015 and March 2016 were identified as belonging to the WGS cluster A and were mainly associated to the MLVA cluster 20–8-10–7-5–4 (Table 2, Figure 3). In terms of food items, only one WGS cluster A isolate from October 2015 was found in raw milk.

The WGS cluster B comprised a total of 35 isolates (20 human and 15 non-human). Two main subclusters were identified; one B3 (mainly associated to the MLVA cluster 17–8-10–7-5–4) was found in relation to cattle, milk and raw-milk cheeses (Reblochon and Morbier) with date of isolation mainly in January 2016 and the other B4 (mainly associated to MLVA cluster 18–8-10–7-5–4) was found in cases with date of isolation mainly in December 2015 and January 2016.

The WGS cluster C was the most prevalent with 123 isolates (88 human and 35 non-human). It was subdivided into nine subclusters (< 5 SNPs), C3 and C_other_ (grouping eight smaller subclusters including C8). All the 35 isolates belonging to the C3 subcluster presented a very limited intra SNP difference (< 2) and a sufficient divergence (> 5 SNPs) to the other eight C subclusters (C_other_), indicating high level of genetic relationship due to a putative common source of contamination. The WGS subcluster C3 was mainly associated with MLVA cluster 18–8-10–7-5–4 and was found in clinical isolates from patients between January and April 2016. This molecular signature was also found in one isolate from Morbier cheese in February 2016 during this investigation. It was found as well in several isolates in Morbier cheeses tested in 2015 through company internal microbiological monitoring system. The WGS subcluster C8 was mainly associated with the MLVA cluster 19–8-10–7-5–3 and harboured isolates from patients at the beginning of 2015. In that period, another *S*. Dublin outbreak had been investigated between February and April 2015 in seven French regions, and was found to be possibly associated with consumption of Reblochon cheese (data not shown; Santé publique France).

Among the non-human isolates, we revealed several other WGS cluster groups (in particular F and K) but no or few linked human isolates (data not shown). The review of the historical human and non-human specimens suggested that WGS B and C cluster isolates have been circulating in France for decades, while WGS cluster A isolates seem to be more recent.

Among the 63 cases for whom a completed questionnaire was available, 58 had had an isolate subtyped by WGS, 55 had had an isolate subtyped by MLVA, and 55 both. Of the 58 cases with WGS subtype, 17 belonged to subcluster C3, 11 to cluster A, and 30 to 14 other subclusters. Of the 55 cases with MLVA pattern, 23 belonged to cluster 18–8-10–7-5–4, 10 to cluster 20–8-10–7-5–4 and 22 to six other clusters.

### Epidemiological investigations

#### Case–case study

Compared with cases belonging to the other WGS clusters, WGS cluster A cases seemed to have consumed Vacherin Mont d’Or cheese more frequently, although this result was not statistically significant (odds ratio (OR): 5.1; 95% confidence interval (CI): 0.9–34). Similarly WGS subcluster C3 cases appeared to be related to Morbier cheese consumption (OR: 3.3; 95% CI: 0.8–15) (Table 3). Compared with cases belonging to other MLVA patterns, MLVA cluster 18–8-10–7-5–4 cases (which frequently coincided with WGS cluster A) seemed to have more often consumed Morbier cheese, however this was not significant (OR: 2.1; 95% CI: 0.5–8.3) and MLVA cluster 20–8-10–7-5–4 cases (which frequently coincided with WGS cluster C3) appeared as consuming more often Vacherin Mont d’Or cheese (OR: 5.1; 95% CI: 0.9–35). No associations were found with other raw-milk cheeses, nor with any other food items.

**Table 3:** Frequency of reported cheese consumption^a^ according to MLVA and whole genome sequencing clusters for the case–case study and the case–control study, *Salmonella* Dublin outbreak, France, November 2015–March 2016 (n cases = 58; n controls = 2,914)

Type of cheese	Cluster cases		Other cases^b^		Controls		Case–case study^c^		Case–control study^d^		Cluster cases		Other cases^b^		Controls		Case–case study^c^		Case–control study^d^	
	n	%	n	%	n	%	OR	95% CI	aOR^e^	95% CI	n	%	n	%	n	%	OR	95% CI	aOR^e^	95% CI
Type of cheese	MLVA 20–8-10–7-5–4 (n = 10 cases)										WGS A (n = 11 cases)									
Vacherin Mont d'Or	7	70	11	31	233	8	5.1	0.9–35	27	6.8–104	7	70	12	32	233	8	5.1	0.9–34	27	6.8–105
Comté	6	67	21	58	1,386	48	1.4	0.3–10.2	NS	NA	6	67	23	59	1,386	48	1.4	0.2–9.8	NS	NA
Gruyère	7	78	26	76	1,841	63	1.1	0.2–13	NS	NA	7	78	28	78	1,841	63	1.0	0.1–12	NS	NA
Camembert	6	67	23	66	756	26	1.0	0.2–7.6	NS	NA	6	67	25	66	756	26	1.0	0.2–7.5	NS	NA
Type of cheese	MLVA 18–8-10–7-5–4 (n = 23 cases)										WGS C3 (n = 17 cases)									
Morbier	12	60	10	42	361	12	2.1	0.5–8.3	11	4.2–29	10	67	12	37	361	12	3.3	0.8–15	14	4.8–42
Goat cheese	9	53	13	54	1,384	47	1.0	0.2–4.0	NS	NA	Not consumed by 50% of the cases						NA	NA	NA	NA
Camembert	12	63	17	68	756	26	0.8	0.2–3.4	NS	NA	11	73	20	62	756	26	1.6	0.4–8.7	NS	NA
Gruyère	13	68	20	83	184	63	0.4	0.1–2.3	NS	NA	10	71	25	81	184	63	0.6	0.1–3.6	NS	NA
Comté	Not consumed by 50% of the cases						NA	NA	NA	NA	7	50	22	65	1,386	48	0.5	0.1–2.3	NS	NA

aOR: adjusted odds ratio; CI: confidence interval; MLVA: Multilocus variable-number tandem repeat analysis; NA: not applicable; NS: did not remain significant in the final logistic regression model; OR: odds ratio; WGS: whole genome sequencing.

^a^ Only cheeses consumed by at least 50% of the cases are included in the analysis; cases and controls could report more than one cheese consumed.

^b^ Cases who belong to the other known subtypes.

^c^ Compares cluster cases with other cases with other known subtypes.

^d^ Compares cluster cases with controls.

^e^ Adjusted for age and sex.

#### Case–control study

After adjustment for age and sex, compared with controls, cases belonging to the WGS cluster A were more likely to have consumed Vacherin Mont d’Or cheese (aOR: 27; 95% CI: 6.8–105) and cases belonging to the WGS subcluster C3 were more likely to have consumed Morbier cheese (aOR: 14; 95% CI: 4.8–42) (Table 3). Compared with controls, cases belonging to the MLVA cluster 18–8-10–7-5–4 were more likely to have consumed Morbier cheese (aOR: 11; 95% CI: 4.2–29) and cases belonging to the MLVA cluster 20–8-10–7-5–4 were more likely to have consumed Vacherin Mont d’Or cheese (aOR: 27; 95% CI: 6.8–104). No other significant associations were found with other raw-milk cheeses, nor with any other food items.

### Food trace-back investigations

We collected 39 (62%) loyalty card numbers from 63 cases. Based on the available information, trace-back investigations were conducted among 10 supermarket brands. Twelve cheese producers were identified as potential origin of the cheeses consumed by the cases. The trace-back investigations linked one Morbier producer and three different Vacherin Mont d’Or producers, to 11, five, four and three cases, respectively. All those producers were located in the same region, i.e. Bourgogne-Franche-Comté (Eastern part of France).

### Food and veterinary investigations

From the 101 non-humans isolates collected in 2015 and 2016, 82 (81%) were collected from milk products (54 from cheese, 27 from milk and one from other dairy), 12 (12%) from animal samples (cattle, meat, faeces) and seven (7%) from environmental samples (milk filter, trough). For the 54 cheese samples, *S*. Dublin was detected in Morbier (n = 37), in Saint-Nectaire (n = 6), in Reblochon (n = 5), in Vacherin Mont d’Or (n = 1) and in other or unknown cheeses (n = 5).

## Discussion

We reported one of the largest *S.* Dublin outbreaks in France in the past few years. Two different bovine raw-milk cheeses, Morbier and Vacherin Mont d’Or, were the most likely vehicles of transmission for this food-borne outbreak. For the present outbreak investigation we used two different typing methods on a large panel of strains (both historical and obtained during the 2015–16 outbreak period, as well as from human and non-human origins). The first method was MLVA, which had already been used in previous investigations of other outbreaks in France [11]. The second, WGS, was used for the first time in the current *S*. Dublin investigation and demonstrated increased capacity to discriminate clusters. We also used two different epidemiological methods: a case–case study that allowed a rapid analysis and identification of suspected sources and a case–control study that was more statistically powerful to confirm the suspected associations.

In this investigation, MLVA was deemed sufficient to identify a link between human cases, food and animal sources. However, MLVA could not distinguish some of the clusters identified by WGS. Our results suggested that at least two outbreaks of *S.* Dublin occurred during the same period, and potentially originated from two different sources. WGS cluster A and subcluster C3 occurred in different periods indicating that they might belong to distinct outbreaks. The retrospective use of WGS also confirmed the occurrence of different *S*. Dublin outbreaks in 2012 [10,11]. High resolution molecular tools like WGS may facilitate linkage of human cases to sources, especially in serotypes with limited intrinsic genetic variation, and may also provide a more detailed picture of the extent and context of the outbreak.

Recruiting controls from an online health cohort survey for an ongoing outbreak investigation was novel in France and served as a pilot to evaluate the suitability of this method in future food-borne outbreak investigations. This method allowed conducting the case–control study in a timely manner with minimum resources, achieving a high response rate [28].

Our investigation pointed towards several cheese producers from the same region as sources of the outbreaks. In this region, an increase in salmonellosis incidence was observed in cattle at the end of summer 2015 (data not shown; Santé publique France). This could explain the increase of contaminated cheese batches in autumn and winter 2015. Veterinary and food investigations were challenging due to (i) high number of possibly implicated processing plants of raw-milk cheeses, and (ii) the high frequency of cheese consumption by cases and the variety of cheeses and places of purchase. It was difficult to identify the exact batches that cases consumed because some cheeses were sold at the deli counter, sliced on demand. Furthermore, the probable low levels of contamination of the implicated cheeses may have led to false negative test results, possibly allowing some contaminated batches to enter the market.

Previous studies indicated that *S*. Dublin is frequently isolated in live cattle, and that cheeses made with unpasteurised milk may be contaminated with *S*. Dublin [8,9]. *S.* Dublin infection is also responsible for substantial losses in the dairy industry [29]. A modelling exercise in Denmark [29] estimated the gross margin losses due to *S.* Dublin infection in a 200-cow stall-herd to be up to EUR 188 per stall annually averaged over the 10-year period following introduction of infection. In that study, relative simple and cheap control measures such as improving calving and colostrum management could lead to significant decreases in prevalence of *S.* Dublin in some herds. In other herds, it was reported that these measures might have to be supplemented by changes in hygiene and feeding practices. It might be worthwhile conducting studies in France to evaluate the impact of recent or future measures on *S*. Dublin prevalence at herd level. In addition, infected cattle might carry chronic and possibly asymptomatic infections while still contributing to onwards transmission by excreting pathogens in faeces [30,31]. SNP-typing based on WGS is a promising tool to monitor the routes and the spread of *S.* Dublin between herds in traditional regions of cheese production, as already reported in previous studies [16,17]. The combination of epidemiological studies in human and non-human sectors and the use of WGS may improve the cost effectiveness of control measures for *S.* Dublin in France, by targeting contaminated herds.

Following the investigations currently reported here, the group of producers of Morbier and Vacherin Mont d’Or cheeses implemented an action plan, including (i) systematic testing for *Salmonella* of batches of Morbier and Vacherin Mont d’Or sold since 1 February 2016, (ii) more regular farm visits by veterinarians, (iii) detection and containment of infected cattle, (iv) expert mission from the Ministry of Agriculture and Food to support milk industry professionals in Bourgogne-Franche-Comté region to identify and recommend better practices for detection and management of contaminated raw-milk products and (v) creation of a working group with experts on *Salmonella* issues from different organisations. The Morbier processing plants union reinforced their sanitary protocols, including more frequent testing of milk.

### Limitations

Our investigations suffered from several limitations. First, cases were interviewed by phone, while controls completed a shorter online questionnaire, which could have led to obtaining exposure data with different degrees of accuracy. To minimise this bias, we used the same questions for cases and controls. Second, age and sex distribution of controls differed from that of the cases. We thus included age and sex in the multivariable analysis to adjust for those characteristics. Third, it was difficult to identify the exact sources of contamination due to probably low levels of contamination by *S.* Dublin of the cheese batches. As the cattle contamination was diffuse, it was difficult to incriminate specific cheese producers in the Bourgogne-Franche-Comté region as sources of contamination. Furthermore, suspected batches of cheese identified through trace-back investigations were no longer available for testing. Then, even if epidemiological investigations were carried out within a very constrained period of time to allow the ad hoc microbiological analyses to be launched to support the generated hypotheses, we had to deal with the impossibility to get through the whole process due to the lack of products.

### Conclusions and recommendations

Microbiological, epidemiological and environmental evidence pointed towards two raw-milk cheeses, Morbier and Vacherin Mont d’Or, as vehicles of the *S*. Dublin infections. The use of MLVA and WGS subtyping methods allowed the identification of different clusters and of the potential vehicles of infection, highlighting the importance of adequate subtyping methods during *Salmonella* outbreaks and the relevance of company internal microbiological monitoring system. As a result, WGS has now been routinely implemented at the French NRC and findings of this multi-disciplinary investigation led to a reinforced control plan for processing plants of raw-milk cheeses to prevent future outbreaks.
